# Synthesis of a New Co Metal–Organic Framework Assembled from 5,10,15,20-Tetrakis((pyridin-4-yl) phenyl)porphyrin “Co-MTPhPyP” and Its Application to the Removal of Heavy Metal Ions

**DOI:** 10.3390/molecules28041816

**Published:** 2023-02-15

**Authors:** Henry Arceo-Ruiz, Elba Xochitiotzi-Flores, Héctor García-Ortega, Norberto Farfán, Rosa Santillan, Susana Rincón, Alejandro Zepeda

**Affiliations:** 1 Facultad de Ingeniería Química, Universidad Autónoma de Yucatán, Periférico Norte Kilómetro 33.5, Tablaje Catastral 13615, Col. Chuburná de Hidalgo Inn., Merida 97203, Mexico; 2 Facultad de Química, Departamento de Química Orgánica, Universidad Nacional Autónoma de México, Ciudad de Mexico 04510, Mexico; 3 Departamento de Química, Centro de Investigación y de Estudios Avanzados del IPN, México, CDMX, Apdo. Postal 14-740, Ciudad de Mexico 07000, Mexico; 4 Tecnológico Nacional de México/I. T. Mérida, Av. Tecnológico km. 4.5 S/N, Merida 97118, Mexico

**Keywords:** metal–organic framework, porphyrin, cobalt, adsorption, lead, kinetic study

## Abstract

The synthesis of a Co metal–organic framework assembled from 5,10,15,20-tetrakis((pyridin-4-yl)phenyl)porphyrin; TPhPyP) “Co-MTPhPyP” is reported. The TPhPyP ligand was synthesized via aldehyde condensation in 28% yield and characterized by ^1^H nuclear magnetic resonance (^1^H NMR), Fourier-transform infrared (FTIR), high-resolution mass spectrometry (HRMS), and UV-visible spectroscopy (UV-vis). Co-MTPhPyP was prepared by the solvothermal method from TPhPyP and CoCl_2_·H_2_O in 55% yield and characterized by X-ray powder diffraction (XRD), FTIR, thermogravimetric analysis (TGA), field-emission scanning electron microscopy with energy-dispersive X-ray (FESEM-EDS), X-ray photoelectron spectroscopy (XPS), and dynamic light scattering (DLS), showing a particle size distribution of 418 ± 58 nm. The sorption properties of the Co-MTPhPyP for the effective removal of Pb(II) and Cu(II) were evaluated in an aqueous medium and Cthe results showed uptake capacities of 383.4 and 168 mg of the metal g^−1^ after 2 h, respectively. Kinetic studies of Pb(II) adsorption by Co-MTPhPyP were adjusted to the pseudo-second-order model with a maximum adsorption capacity of 458.8 mg g^−1^ at 30 min of exposition.

## 1. Introduction

Metal–organic frameworks (MOFs) are a class of porous crystalline materials with large specific surface area, porosity, and thermal stability, among other suitable properties depending on the metal ions or clusters and the organic linkers. These characteristics make them excellent candidates for various applications in many important areas, which include catalysis, electrode fabrication, drug carriers, adsorption of gases and organic pollutants and also for the removal of heavy metals present in water [1,2,3]. In this sense, a number of MOFs have been successfully used as metal ion adsorbent materials in aqueous medium [4,5]; for example, Tahmasebi et al. [6] reported the mechanochemical synthesis of zinc-based MOFs (TMU-4, TMU-5, and TMU-6) with azine linkers and imine groups as adsorbents of Cu(II) and Pb(II), with a maximum adsorption capacity to remove Pb(II) and Cu(II) of 251 and 62 mg g^−1^, respectively. Likewise, Bakhtiari and Azizian applied MOF-5 as Cu(II) ion adsorbent, reporting rapid adsorption and reaching equilibrium after 30 min, with a maximum adsorption capacity of 290 mg g^−1^ at pH 5.2 and 45 °C [7]. Despite these results, the application of MOFs as sorbent material remains controversial due to evidence reported at both computational and experimental level concerning the poor structural stability of some MOF’s such as MOF-5, MIL-101, and HKUST, which undergo degradation by the exchange of linkers or hydrolysis when exposed to water in both liquid and vapor phases [8,9]. Rivera et al. [10], for example, used MOF-5 for the removal of Pb(II) reporting an adsorption capacity of 658.5 mg g^−1^; however, they also evidenced the disruption of the structure of MOF-5 in an aqueous medium, due to a possible interaction of the benzene dicarboxylate ion (BDC^2−^) with Pb(II), caused by the coordination of the Zn^2+^ contained in the Zn_4_O complexes, with the oxygen atoms of the water through nonbonded interactions (electrostatic and van der Waals). This adverse reaction could generate environmental and human health problems, due to the release of these compounds into the aqueous medium. For these reasons, it becomes important to develop novel MOF-type compounds that contain ligands and metal clusters structurally stable in aqueous medium to avoid the release of their structural components and thus provide sustainable materials that can be applied to the elimination of contaminants present in water and wastewater.

In this sense, various studies on MOFs synthesized from porphyrins have shown surprising stability in the presence of water. For example, Sadeghi et al. [11] reported the preparation of a metal–organic Zn-PMOF structure using tetrakis(4-carboxyphenyl)porphyrin as a ligand for the photoreduction of CO_2_ to CH_4_ with water vapor. This Zn-PMOF showed high stability and reuse, since there were no significant losses of the photocatalytic activities or changes in the structure of the photocatalyst evidenced through the UV-vis and FTIR spectra obtained upon contact with water. Similarly, Deiber et al. [12] reported great stability in water of a MOF made up of Zr_6_(µ_3_-OH)_8_(OH)_8_(CO_2_)_8_ “MOF PCN-225,” where (CO_2_)_8_ is the fraction corresponding to the carboxylate groups of the *meso*-tetra(4-carboxyphenyl) porphyrin used as ligand. This MOF showed high structural stability in the presence of water, which was preserved after 9 months in contact with water. However, despite the stability of MOF porphyrins, there are few studies on their capacity for the adsorption of heavy metals in aqueous media.

To date, several groups have reported the use of porphyrins for the construction of different nanomaterials with the aim of increasing or conferring sorbent capacity towards various heavy metals. For example, Behbahani et al. [13] reported the application of a nanostructured material from fructose doped with tetracarboxyphenyl porphyrin (TCPP) for the removal of heavy metals in an aqueous medium, reporting maximum adsorption capacities of 81.6, 48.3, 41.4, and 53.6 mg g^−1^ for Cd(II), Ni(II), Cu(II), and Fe(III) ions, respectively, with 15 desorption–adsorption cycles without significant loss in adsorption capacity. Similarly, Bakhshayesh and Dehghani [14] reported the synthesis of magnetite nanocomposites (MPNC) modified with TCPP porphyrin as a magnetic adsorbent of Pb(II), Cd(II), and Hg(II) ions in aqueous medium, finding that the addition of porphyrin increased the lead adsorption capacity and was responsible for the removal of cadmium and mercury ions in 45%, 15%, and 10%, respectively.

On the other hand, there are few studies on the use of MOFs with Co as cluster as adsorbent material in aqueous medium, even though these types of MOFs have been used successfully in different areas (catalysis, manufacture of electrodes, etc.), showing in some cases high structural stability [15,16]. In addition, it has been reported that Co MOFs in some cases do not cause toxicity on microorganisms and exhibit a low cytotoxic effect towards in a liver cell line (LO2) [17,18]. For example, Ordaz et al. [17], through microrespirometric studies on activated sludge in the presence of a cobalt-based organic metal compound (MOF-Co), found that this material did not inhibit oxygen consumption of the activated sludge.

Thus, the main objective of this study was to synthesize a Co-based porphyrin MOF (Co-MTPhPyP) from a novel porphyrin (5,10,15,20-tetrakis((pyridin-4-yl)phenyl)porphyrin), which exhibits an effective adsorption capacity for the removal of heavy metals such as (Pb(II) and Cu(II)) in an aqueous medium.

## 2. Results and Discussion

### 2.1. Synthesis and Characterization of Ligand TPhPyP

**Figure 1:**
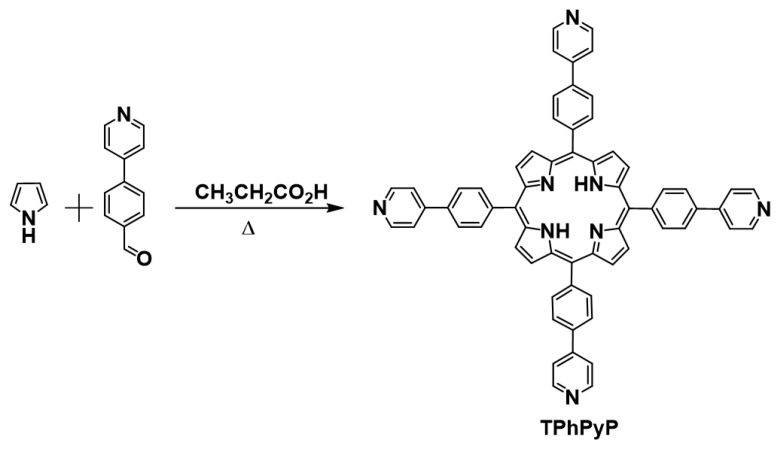
Synthetic route for the preparation of porphyrin TPhPyP.

Porphyrin TPhPyP was prepared by reaction of pyrrole with 4-(4-formylphenyl)pyridine in 28% yield using a previously described methodology before (Figure 1). The structure of the molecule was confirmed by HRMS, which showed a peak at 923.36 g mol^−1^, corresponding to the molecular weight (Figure S1) and spectroscopically characterized by ^1^H NMR complemented by FTIR and UV-vis. The characteristic signals in ^1^H NMR that evidenced the formation of the desired product are the broad signal to −2.7 ppm associated with the NH proton of the porphyrin core, and the singlet at 8.94 ppm corresponding to the protons at the *β*-pyrrolic position of the porphyrin ring. In the aromatic region, the two double doublets at 8.84 ppm with *J* = 4.5, 1.6 Hz and 7.85 ppm with *J* = 4.5, 1.6 Hz were assigned to the pyridine ring and the other two doublets to the protons of the phenyl ring at 8.37 ppm with *J* = 8.17 Hz and 8.08 ppm with *J* = 8.17 (Figure S2).

**Figure 2:**
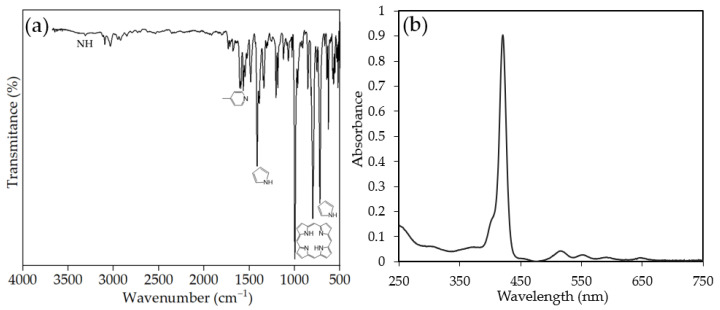
(**a**) FTIR spectra of TPhPyP and (**b**) UV-vis.

FTIR analysis of TPhPyP (Figure 2a) showed the weak band at 3313 cm^−1^ attributed to the NH (ν-NH), the band at 1593 cm^−1^ was an assigned to the pyridine skeletal stretching. The two medium bands at 1539 cm^−1^ were assigned to the pyridine skeletal stretching and two medium bands at 1539 cm^−1^ and 1473 cm^−1^ correspond to C=C and C=N stretching bands, respectively. The strong band at 966 cm^−1^ corresponds to deformation of the porphyrin ring and the strong band at 798 cm^−1^ was attributed to pyrrole ring deformation [19,20]. The UV-vis spectra data for the TPhPyP were obtained in CHCl_3_ and showed a typical electronic spectrum of *meso-*substituted porphyrin with a sharp short band at 422 nm and four Q-bands at 519, 554, 594, and 647 nm (Figure 2b).

### 2.2. Characterization of Co-MTPhPyP

#### 2.2.1. FTIR Analysis

**Figure 3:**
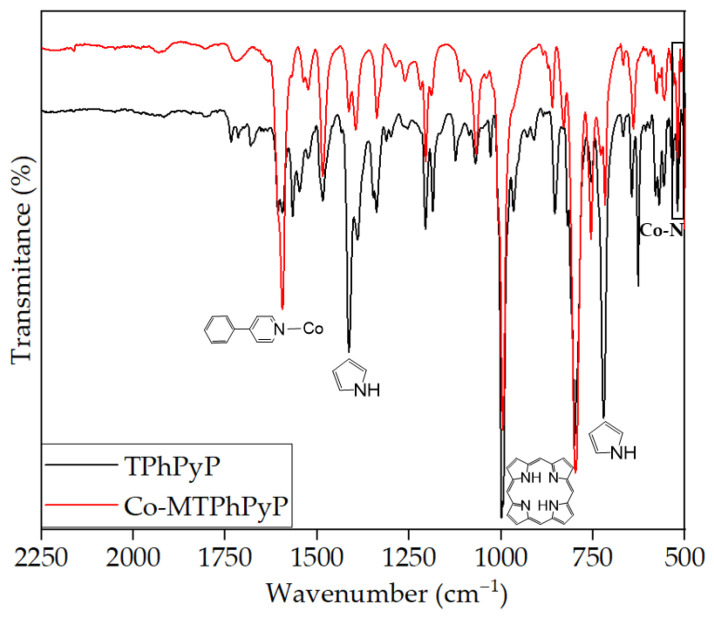
FTIR spectra of porphyrin TPhPyP (black) and Co-MTPhPyP (red).

The infrared spectrum of Co-MTPhPyP (Figure 3) confirmed the presence of the band assigned to pyridine around about 1592 cm^−1^ with satellite band at 1605 cm^−1^ for CoTPhPyP according to Tomita et al. [21]. The intense band for the TPhPyP ligand (1416 cm^−1^) attributed to the superposition of pyridine vibrations in shifted to 1384 cm^−1^ upon coordination with cobalt and the bands at 998 cm^−1^ and 800 cm^−1^ for to the deformation of the porphyrin ring and the deformation of the pyrrole nucleus, respectively, remained unaffected. The new band at 760 cm^−1^ was assigned to Co-Cl stretching vibrations. The characteristic band due to the deformation of the pyridine nucleus was shifted from 798 cm^−1^ in the ligand to 721 cm^−1^ after coordination with cobalt. In addition, the new weak bands at 521 cm^−1^ and 468 cm^−1^ were attributed to Co-N vibrations, and corroborate coordination of cobalt at the *meso* position of TPhPyP, in agreement with reports for similar structures [22,23,24]. These data confirmed the formation of Co-MTPhPyP.

#### 2.2.2. XPS Analysis

**Figure 4:**
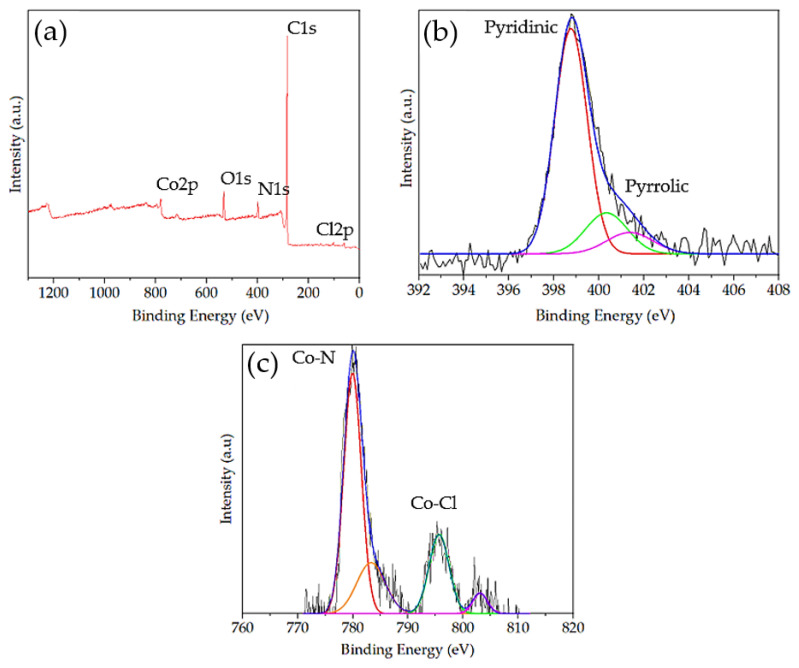
XPS spectra of Co-MTPhPyP (**a**), N 1s spectra (**b**) and Co 2p spectra (**c**).

XPS spectra confirmed the presence of C, N, Co, and Cl in Co-MTPhPyP (Figure 4a). The signals of C 1s peak at 285.03 eV, Cl 2p at 199.43 eV, N 1s at 399.21 eV and Co 2p at 780.16 eV and 795.88 eV were observed. The surface atomic percentages of C, N, O, Cl, and Co are 85.91, 5.4, 6.52, 0.51, and 1.66%, respectively. The high-resolution N1s spectrum (Figure 4b) indicates the presence of three predominant nitrogen signals, which include pyridinic N at 398.76 eV, pyrrolic N at 400.32 eV, and quaternary N at 401.5 eV. The signal at 398.76 eV includes the contribution of the nitrogen–cobalt (N-Co) bond due to the small difference between the binding energy of Co-N and N-pyridinic [25,26]. The de-convulsion of Co signals is presented in Figure 4c. Signals at 779.99 eV and 795.7 eV were assigned to Co peaks 2p3/2 and 2p1/2 with zero valence. Furthermore, several authors show that signals around 780 eV and 795 eV are attributed to the binding energy of Co-N and Co-Cl, respectively [25,26,27].

#### 2.2.3. FESEM-EDS and DLS Analysis

**Figure 5:**
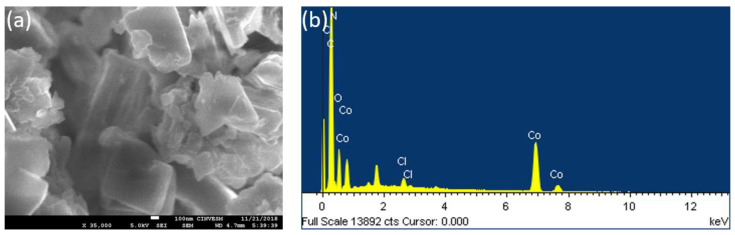
Micrography SEM (**a**) and EDS (**b**) for Co-MTPhPyP.

The morphology and chemical composition of Co-MTPhPyP were determined by FESEM equipped with an EDS system. The micrographs obtained show that the morphology of Co-MTPhPyP is homogeneous and corresponds to a rectangular prism (Figure 5a). Further confirmation of the presence of Co in the structure of Co-MTPhPyP was obtained from EDS analysis. In Figure 5b, the EDS pattern shows a clear Co signal indicating that Co incorporation was successfully carried out in Co-MTPhPyP. In addition, the totality of Co in the structure is 1.53% by atomic weight, agreeing with what was obtained by the XPS studies (1.66%). Finally, DLS analysis using the Zetasizer Nano (Malvern) shows that the average particle size of the Co-MTPhPyP dispersed in water is 478.5 ± 163 nm in diameter.

#### 2.2.4. XRD Analysis

**Figure 6:**
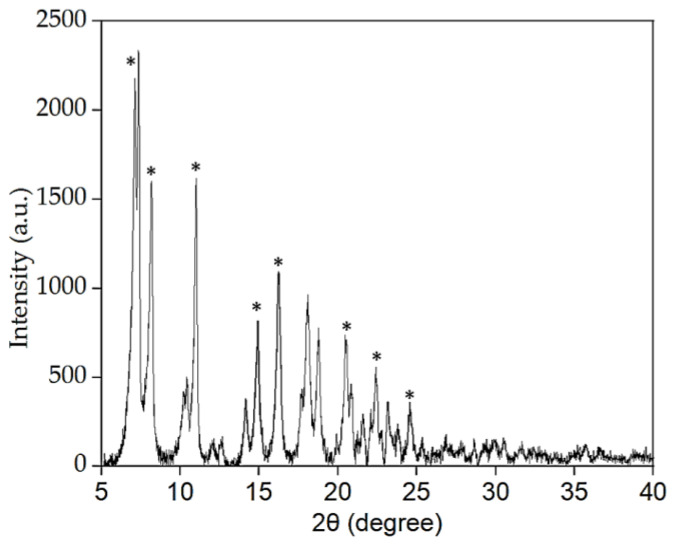
XRD pattern of Co-MTPhPyP. * Signals similar toy Sengupta et al. [24].

The XRD powder pattern (Figure 6) confirmed a crystalline structure of Co-MTPhPyP by showing strong and well-defined diffraction peaks at 2θ: 7.15, 8.17, 11.02, 14.89, 16.22, 20.45, 22.41, and 24.59, which are characteristic of porphyrin-based MOF [22,24]. Moreover, XRD powder pattern is similar to that reported by Sengupta et al. [24] with a 2D MOF [Cu(TPyP)Cu_2_(O_2_CCH_3_)_4_] (Cu-MOF), using 5,10,15,20-tetra-4-pyridyl-21H,23H-porphine (porphyrin, H_2_TPyP) linkers.

**Figure 7:**
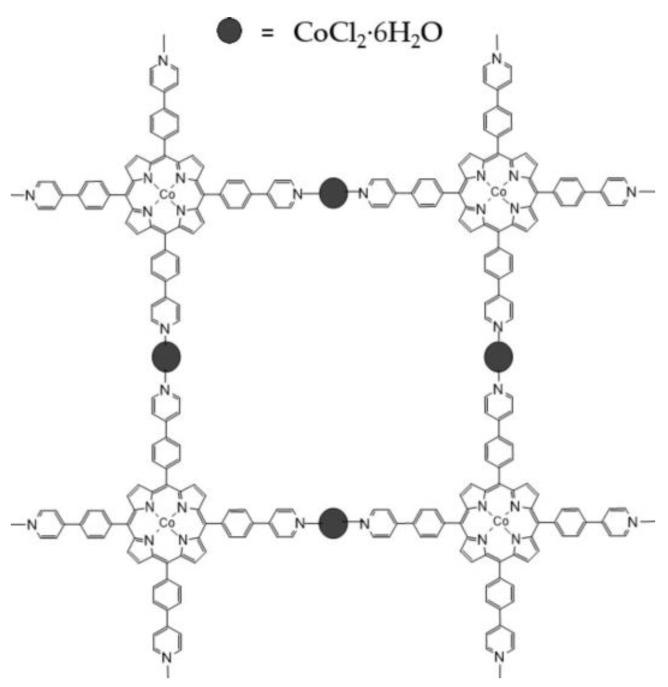
2D structure of Co-MTPhPyP.

In conclusion, considering the FTIR, XRD and XPS analysis, a possible 2D structure of Co-MTPhPyP is shown in Figure 7, in accordance with the reported by Sengupta et al. [24] with a Cu-MOF. Suggesting the form 2D network structure with a face-on arrangement in accordance with Tomita et al. [21].

### 2.3. Adsorption of Co-MTPhPyP for Ions Pb(II) and Cu(II)

The results for the adsorption of the heavy metals in an aqueous medium using Co-MTPhPyP as adsorbent material are presented in Table 1, obtaining an adsorbent capacity for the removal of Pb(II) and Cu(II) of 383.4 mg g^−1^, and 168 mg g^−1^ after 2 h of reaction respectively. Similar results have been obtained in various previous works when using MOFs as adsorbent material for Pb(II) and Cu(II) (Table 1). For example, Xu et al. [28] studied the adsorption capacity of nanosheets of MOF Zn(Bim)(OAc) based on Zn, benzimidazole, and acetate ion to capture heavy metals in an aquatic system, through isotherms of adsorption obtaining a maximum adsorption capacity of 253.8 mg g^−1^ for Pb(II) and 335.57 mg g^−1^ for Cu(II). In turn, Tahmasebi et al. [6] evaluated the adsorption capacity of Pb(II) and Cu(II) in water using various Zn-based MOFs (TMU-4, TMU-5, and TMU-6) functionalized with azine, through adsorption isotherms, obtaining values of 251 mg g^−1^ and 62 mg g^−1^, respectively. Furthermore, Lu et al. [29], used MOFMIL-101(Fe)/GO based on Fe with graphene oxide, to carry out the adsorption of Pb(II) in an aqueous medium through isotherm studies, obtaining a maximum adsorption capacity of 128.6 mg g^−1^. In another work, Li et al. [30] obtained a maximum adsorption capacity of 87.37 mg g^−1^ of Pb(II) in an aqueous solution using zero-valence iron supported on a zeolite (Z-NZVI). Mousavi et al. [31] investigated the removal of Cu(II) from an aqueous solution using a magnetic chitosan compound based on SBA-15 modified with amine groups (Fe_2_O_3_@SBA-15 − CS − APTMS) with which through the methodology of response surfaces and adsorption isotherms obtained a maximum adsorption capacity of 107.3 mg g^−1^ of Cu(II). As can be seen, the Co-MTPhPyP prepared in our study presented capacities similar to other MOFs and various materials used in the adsorption of Pb(II) and Cu(II). However, Co-MTPhPyP was neither functionalized nor evaluated under adsorption isotherm studies showing the largest adsorption capacity of the adsorbent materials reported in Table 1, through the evaluation of adsorption isotherms. In this way, the preliminary results obtained in our study are encouraging, since evaluation of Co-MTPhPyP in adsorption isotherms in the presence of Pb(II) and Cu(II) gave results similar to those reported by Rivera et al. [10] and Xu et al. [28], respectively (Table 1).

#### Kinetic Study for the Adsorption of Pb(II) with Co-MTPhPyP

**Figure 8:**
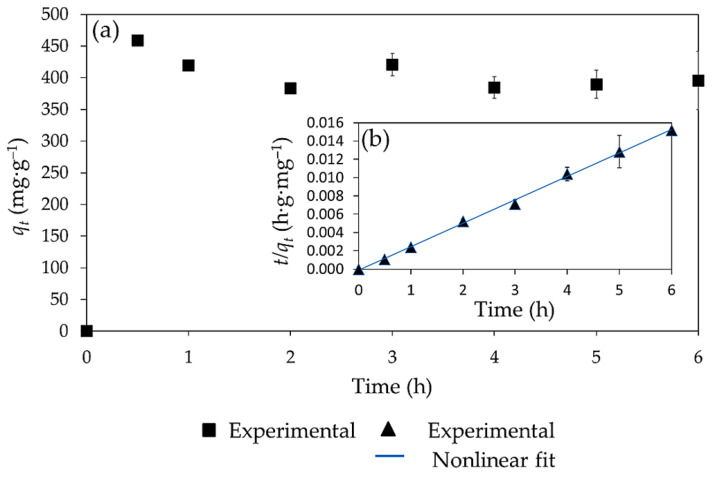
Kinetic study (**a**) and pseudo-second-order kinetic models (**b**) for the adsorption of Pb(II) on Co-MTPhPyP.

The data for the kinetic study of Pb(II) adsorption are presented in Figure 8. The results show that the maximum adsorption capacity was reached in a short time (30 min) with an adsorption capacity of 458.8 mg g^−1^ (Figure 8a). Likewise, it was possible to observe that longer exposure time caused a desorption effect of up to 78 mg g^−1^ after 2 h of contact, possibly due to competition and interactions with H^+^ protons. Therefore, optimal contact time to carry out the adsorption of Pb(II) was 30 min. This result is consistent with some recent research. For example, Yu et al. [34] using a tetracarboxyphenyl porphyrin (TCCP) doped with a magnetic compound, obtained an adsorption capacity of 384 mg g^−1^, in 6 min at an initial concentration Pb(II) concentration of 16 mg L^−1^. In another study, Liu et al. [35] functionalized cotton fibers with tetramethylpyridine porphyrin (TMPyP) for the adsorption of Cd(II), obtaining an adsorption capacity of 97.1 mg g^−1^ after 2 min of exposure. This indicates that the adsorption capacity of Co-MTPhPyP towards Pb(II) could be improved using a shorter adsorption time. Moreover, the analysis FTIR, showed that characteristic signals of Co-MTPhPyP (1592 cm^−1^, 1605 cm^−1^, 1384 cm^−1^, 998 cm^−1^, 798 cm^−1^, 521 cm^−1^) after the adsorption process (6 h of reaction) were not significantly modified (Figure S3).

Finally, through the pseudo-first- and pseudo-second order adsorption kinetic models, it was possible to observe that the Pb(II) adsorption kinetics has a pseudo-second-order behavior with a correlation coefficient R^2^ of 0.998 (Figure 8b) compared to the pseudo-first-order model with an R^2^ of 0.001 (Table S1). The pseudo-second-order kinetic model suggests that the adsorption process is carried out by chemisorption through the sharing of valence forces or in the exchange of electrons between the metal ions and the adsorbent [36]. This behavior agrees with other studies on MOFs in the adsorption of heavy metal ions [6,10,29,32].

In this way, the preliminary results obtained through our study can be encouraging, since through the evaluation of Co-MTPhPyP in adsorption isotherms in the presence of Pb(II) and Cu(II), results could be obtained equal to or higher than those reported by Rivera et al. [10] and Xu et al. [28], respectively (Table 1). Therefore, more studies are necessary to be able to evaluate the adsorbent capacity of heavy metals through Co-MTPhPyP.

## 3. Materials and Methods

### 3.1. Synthetic Procedures

The chemical reagents pyrrole (reagent grade, 98%, Sigma-Aldrich), 4-(4-formylphenyl)pyridine (reagent grade, 97%, Sigma-Aldrich), propionic acid (reagent grade, 99.5%, Sigma-Aldrich), cobalt(II) chlorate dihydrate (reagent grade, 97%, Sigma-Aldrich), and dimethylformamide “DMF” (reagent grade, anhydrous, 99.8%, Sigma-Aldrich) were used without further purification.

The TPhPyP ligand was characterized by ^1^H nuclear magnetic resonance (^1^H NMR) spectra obtained at 500 MHz, on a Bruker Advance AVANCE DMX-500 spectrometer using deuterated chloroform (CDCl_3_) as solvent. Chemical shifts (δ) are given in ppm and all coupling constants (J) are reported in Hertz. Fourier-transform infrared (FTIR)q spectra were measured on a Perkin Elmer Spectrum 400 FTIR/FT-FIR spectrometer using KBr pellets (units are reciprocal centimeters). UV-vis spectroscopy (UV-vis) spectra were recorded on a PerkinElmer Lambda 900 spectrophotometer using 1.0 cm cuvettes. High-resolution mass spectrometry (HRMS) spectra were obtained on an AB SCIEX 4800 Plus MALDI TOF/TOF analyzer.

The morphology and structure of Co-MTPhPyP was determined using field-emission scanning electron microscopy with energy-dispersive X-ray (FESEM-EDS) (JEOL-JSM-35). Co-MTPhPyP samples were characterized before and after adsorption by IR with a Thermo Nicolet Nexus 670 FTIR spectrometer using KBr pellets and by attenuated total reflectance–Fourier-transform infrared spectroscopy (ATR-FTIR) on a FTIR Thermo Scientific™ Nicolet™ iS™ in the 4000−500 cm^−1^ wavenumber range. X-ray photoelectron spectroscopy (XPS) was determined on a Thermo Scientific K-alpha spectrometer. X-ray powder diffraction (XRD) patterns were obtained in a Bruker D8 Avance instrument using CuKα (λ = 1.5405 Å) radiation, a scan speed of 1°/min and a step size of 0.02° in 2*θ*. The particle size was measured by dynamic light scattering (DLS) using a Zetasizer Nano ZS (Malvern) dispersing Co-MTPhPyP particles in distilled water (pH = 6.4).

### 3.2. Synthesis of 5,10,15,20-Tetrakis((Pyridin-4-yl) Phenyl)Porphyrin (TPhPyP)

In a round-bottomed flask equipped with a straight condenser and a round magnetic stirrer were placed 12 mL of propionic acid and heated to reflux followed by addition of 4-(4-formylphenyl)pyridine (500 mg, 2.73 mmol) and pyrrole (183.1 mg, 2.73 mmol). The reaction mixture was refluxed for 30 min [37,38], cooled to room temperature and the crude product was filtered and washed with methanol. The resulting solid was purified by column chromatography (silica gel) using 7:3 hexane:dichloromethane as eluent, yielding a purple solid of TPhPyP that was obtained in 28% yield (141 mg, 0.153 mmol). ^1^H NMR [400 MHz, CDCl_3_] (δ, ppm): 8.94 (s, 2H, H-β), 8.84 (dd, *J* = 4.5, 1.6 Hz, 2H, H-7), 8.37 (d, *J* = 8.1 Hz, 2H, H-2), 8.08 (d, *J* = 8.1 Hz, 2H, H-3), 7.85 (dd, *J* = 4.5, 1.6 Hz, 2H, H-6), −2.70 (s, 2H, NH). Anal. calcd. for C_64_H_43_N_8_ m/z 923.36 MS: m/z 923.36 ([M+H]^+^) error: 0.030 ppm. UV-Vis (CHCl_3_): λ_max_ = 423 nm (Soret band), 519, 554, 594, 647 (Q bands). FTIR: 3313 (w), 3030 (w), 1593 (s), 1606 (sh), 1539 (w), 1473 (w), 1396 (m), 1348 (m), 1014 (w), 966 (s), 798 (vs), 742 (sh), 721 (vs).

### 3.3. Synthesis of Co-MTPhPyP

In a round-bottom flask were placed TPhPyP (50 mg, 0.054 mmol) and CoCl_2_·6H_2_O (64.26 mg, 0.27 mmol) in 10 mL of DMF under magnetic stirring for 20 min. The reaction mixture was then transferred to a Teflon-lined stainless-steel autoclave and heated to 130 °C for 10 days. After cooling to room temperature, the product was collected by filtration, washed with ethanol and distilled water several times. A purple crystalline powder with violet microcrystals was obtained in 55% yield (27.5 mg) based on TPhPyP.

### 3.4. Adsorption Capacity of Co-MTPhPyP on Pb(II) and Cu(II)

To demonstrate that Co-MTPhPyP is capable of adsorbing metal ions, 1000 mg L^−1^ standard solutions of Pb(NO_3_)_2_·2H_2_O and CuSO_4_·6H_2_O were prepared. From the standard solution, 100 mL solutions with a concentration of 50 mg L^−1^ of each metal were prepared, followed by addition of 6.5 mg of Co-MTPhPyP for each of the experiments. The pH of the solutions was set to 5 ± 0.1 by adding 0.1 M sodium hydroxide or hydrochloric acid. All the experiments were carried out in triplicate at 25 °C. After 2 h of exposure, the samples were centrifuged at 15,000 rpm for 15 min, to recover the Co-MTPhPyP with the metal removed from the aqueous solution. From the solution, a 15 mL aliquot was taken and filtered through a 0.45 µm pore membrane, to carry out the quantification of heavy metals by flame atomic adsorption equipment (Analyst 800 Perkin Elmer equipment). The adsorption capacity (*q* in mg L^−1^) of Co-MTPhPyP for metal ions was calculated following Equation (1) [39].

(1) $$q=\frac{\left(C_{0}-C_{f}\right)}{m}V$$

where *C_0_* and *C_f_* are the initial and final concentration of the metal (mg L^−1^), respectively, *V* is the volume of the solution (L), and *m* is the weight of Co-MTPhPyP (g).

The equilibrium adsorption capacity (*q_e_*) of Pb(II) per unit mass of MOF was obtained using Equation (2); Equation (3) was used to calculate the adsorption capacity at a specific time (*q_t_*) [40].

(2) $$q_{e}=\frac{C_{0}-C_{e}}{m}V$$

(3) $$q_{t}=\frac{C_{0}-C_{t}}{m}V$$

### 3.5. Kinetic Analysis for the Sorption of Pb(II) by MTPhPyP-Co

To obtain the adsorption equilibrium time for Pb(II), an adsorption kinetics study was carried out. For this, 6.5 mg of MTPhPyP-Co was used, at a concentration of 50 mg L^−1^ of Pb(II) as a metal ion, and pH-5 adjusted with NaOH and HCl. Samples were taken at specific time intervals (0, 0.5, 1, 2, 3, 4, 5, and 6 h) during the adsorption process and analyzed by atomic adsorption equipment. Kinetics data was analysis by the pseudo-first order and pseudo-second-order kinetic models with the aim to explain if adsorption was carried out by physisorption and chemisorption respectively [41,42], expressed in Equations (4) and (5) [40], where *q_e_* and *q_t_* are the adsorption capacity (mg g^−1^) in equilibrium and in contact at time *t*, respectively, C_0_, C_t_, and C_e_ are the concentrations of Pb(II) (mg L^−1^) contained in the original solution, after time *t* and equilibrium, respectively, *V* is the volume of the solution (mL), and *m* represents the weight of Co-MTPhPyP (g)

(4) $$\ln\left(q_{e}-q_{t}\right)=\;\ln q_{e}-k_{1}t$$

(5) $$\frac{t}{q_{t}}=\frac{1}{k_{2}q_{e}{}^{2}}+\frac{t}{q_{e}}\;$$

where k_1_ (1 min^−1^) y k_2_ (g mg^−1^ min^−1^) are the constants in these two models.

## 4. Conclusions

Herein, we described the synthesis of a novel metal–porphyrin framework with cobalt (Co-MTPhPyP). Co-MTPhPy showed an adsorbent capacity of Pb(II) and Cu(II) of 383.4 y 168 mg of metal g^−1^ after 2 h of contact. Kinetic studies of Pb(II) adsorption with Co-MTPhPy showed a maximum adsorption capacity of 458.8 mg g^−1^ at 30 min of exposition by chemisorption according to the pseudo-second-order model. Co-MTPhPyP could be considered an excellent alternative for the removal of Pb(II) and Cu(II) from water and wastewater. However, more studies about its possible reversibility of Co-MTPhPyP as well as adsorption isotherm studies are necessary to understand the mechanism and adsorption maximum capacity of heavy metal ions in water by Co-MTPhPyP.

## Figures and Tables

**Table 1:** Comparative table of different MOFs and compounds used for the adsorption of Pb(II) and Cu(II).

MOF	Adsorption Capacity (mg g^−1^)	Reference
MOF-5 (Zn)	658 [Pb^2+^] *	[10]
MIL-101 (Fe)/GO	128.6 [Pb^2+^] *	[29]
Zn(Bim)(OAc)	253.8 [Pb^2+^] *	[32]
	335.6 [Cu^2+^] *	
MOF TMU-6 (Zn)	224.0 [Pb^2+^] * 60.0 [Cu^2+^] *	[6]
MOF TMU-4 (Zn)	237.0 [Pb^2+^] * 62.0 [Cu^2+^] *	
MOF TMU-5 (Zn)	251.0 [Pb^2+^] * 57.0 [Cu^2+^] *	
Co-MTPhPyP	383.4 [Pb^2+^]	This work
	168.0 [Cu^2+^]	
**Other Compounds**		
PAMAM-SBA-15 (polyamidoamine-SBA-15)	242.4 [Pb^2+^] *	[33]
	110.5 [Cu^2+^] *	
Zeolite-Nanoscale Zero Valent Iron (Z-NZVI)	85.37 [Pb^2+^] *	[30]
Fe_2_O_3_@SBA-15-CS-APTMS	107.3 [Cu^2+^] *	[31]

[*] Maximum adsorption capacity obtained in isotherm studies.

## Data Availability

Not applicable.

## Supplementary Materials

The following supporting information can be downloaded at https://www.mdpi.com/article/10.3390/molecules28041816/s1. Figure S1: HRMS spectrum of TPhPyP; Figure S2: ^1^H NMR spectrum of TPhPyP, Figure S3: FTIR spectra of TPhPyP (a) before adsorption and (b) after absorption of Pb(II); Table S1: Kinetic data of the pseudo-first and pseudo-secondo-order absorption Pb(II) by Co-MTPhPyP.
