# Supplementary material for: Synthesis of a New Co Metal–Organic Framework Assembled from 5,10,15,20-Tetrakis((pyridin-4-yl) phenyl)porphyrin “Co-MTPhPyP” and Its Application to the Removal of Heavy Metal Ions
Source: Molecules. 2023 Feb 15;28(4):1816. doi: 10.3390/molecules28041816 (PMC9964701; doi:10.3390/molecules28041816)
Supplement: Supplementary file 1 [file molecules-28-01816-s001.zip › molecules-2169573-supplementary.pdf]

Supplementary Materials

Supplementary Figure S1. HRMS spectrum of TPhPyP

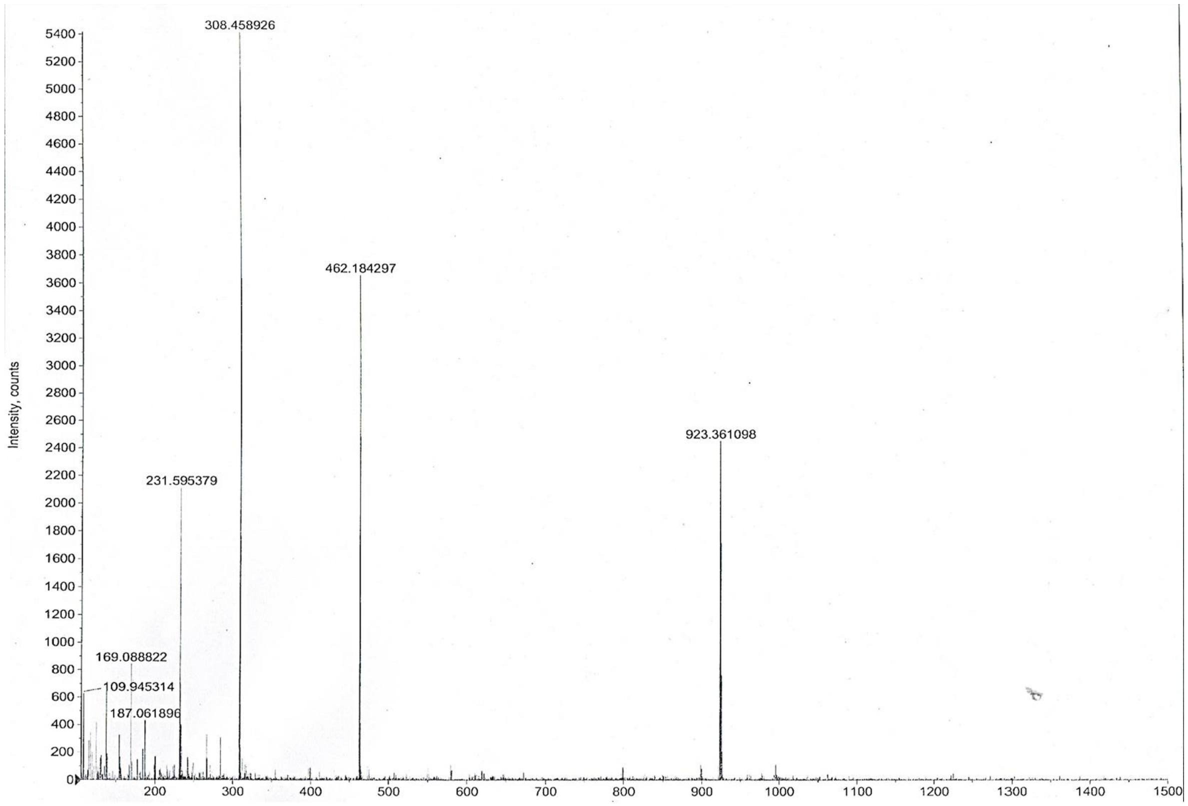

Supplementary Figure S2. ^{1}H NMR spectrum of TPhPyP

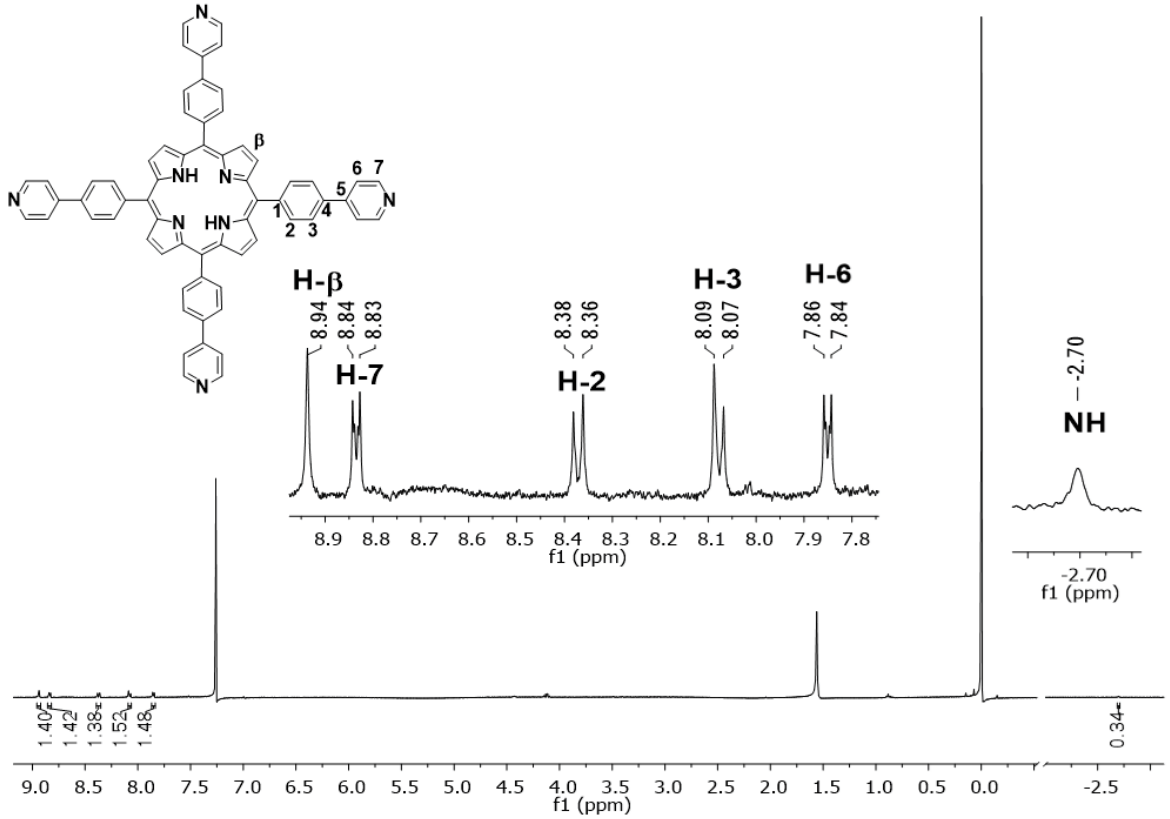

Supplementary Figure S3. FTIR spectra of TPhPyP a) before adsorption and b) after absorption of Pb(II).

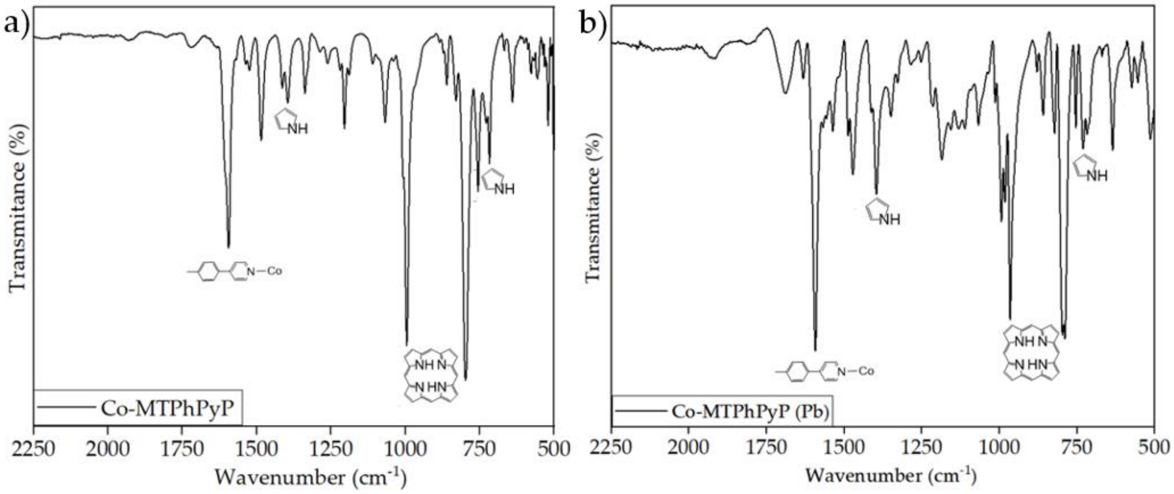

Supplementary Table S1. Kinetic data of the pseudo-first and pseudo-secondo-order absorption Pb(II) by Co-MTPhPyP .

**Concentration (mg/L)**	**Pseudo-first-order**			**Pseudo-second-order**		
	K_1 (g·mg^{-1}·min^{-1})	q_e (mg·g^{-1})	R^2	K_2 (g·mg^{-1}·min^{-1})	q_e (mg·g^{-1})	R^2
50	-0.549	0.98	0.001	-0.033	384.62	0.998
